# A Modular Perfusion Bioreactor Platform for Simulating Bone Regeneration and Fracture Healing: Integrating Mechanical Loading and Dual Perfusion for Advanced In Vitro Models

**DOI:** 10.1002/adhm.202502492

**Published:** 2025-08-15

**Authors:** Moritz Pfeiffenberger, Alexandra Damerau, Johannes Plank, Adel Ahmed, Mario Thiele, Jan Saam, Paula Hoff, Timo Gaber, Frank Buttgereit

**Affiliations:** ^1^ Department of Rheumatology and Clinical Immunology Charité – Universitätsmedizin Berlin Corporate Member of Freie Universität Berlin and Humboldt‐Universität zu Berlin 10117 Berlin Germany; ^2^ German Rheumatism Research Centre (DRFZ) Berlin A Leibniz Institute 10117 Berlin Germany; ^3^ ASML Berlin 12347 Berlin Germany; ^4^ Julius‐Wolff‐Institute Charité – Universitätsmedizin Berlin, corporate Member of Freie Universität Berlin, and Humboldt‐Universität zu Berlin 13353 Berlin Germany; ^5^ Heidolph Scientific Products GmbH 91126 Schwabach Germany; ^6^ MVZ Endokrinologikum Berlin am Gendarmenmarkt 10117 Berlin Germany

**Keywords:** mechanical loading, modular bioreactor platform, tissue engineering

## Abstract

Recent advancements in tissue engineering have led to sophisticated in vitro models that better replicate physiological conditions. Bone regeneration remains a key research area due to its complex remodeling and biomechanical properties. Traditional models often fail to capture these dynamics, limiting their translational potential. Here, a modular bioreactor platform designed to simulate bone homeostasis and disease states with integrated mechanical load simulation is presented, featuring a 3D‐printed microfluidic chamber, dynamic dual perfusion, and a mechanical compression device, enabling precise control of environmental parameters via a web interface. Applied to an in vitro fracture healing model, the setup prolonged viability by facilitating the inflammatory‐to‐anti‐inflammatory transition. Additionally, the setup allowed for generating functional bone models through controlled mechanical stimulation, revealing mechanobiological insights. The dual perfusion approach further enhanced composite tissue incubation. This system advances in vitro tissue modeling by combining perfusion with mechanical stimulation, improving nutrient delivery, mechanotransduction, and scalability. It holds promise for preclinical research, drug testing, and regenerative medicine, bridging the gap between static in vitro models and physiologically relevant conditions.

## Graphical abstract

A modular bioreactor platform was developed in order to replicate bone homeostasis and pathology, thereby integrating mechanical loading, dynamic perfusion, and a 3D‐printed microfluidic chamber. With precise control of environmental parameters and dual perfusion for composite tissue models, the system enhances physiological relevance for studying bone regeneration and fracture healing under controlled, dynamic conditions.

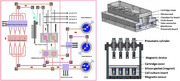

## 1 Introduction

Research in the field of tissue engineering and regenerative medicine has been developing rapidly in recent years.^[^ ^1^ ^]^ Therefore, there is increasing interest in sophisticated in vitro models, oftentimes realistically reflecting physiological conditions.^[^ ^2^ ^]^ Such models are essential to increase the validity and predictability of experimental studies, making them indispensable for understanding complex biological processes and developing new therapeutic approaches and strategies.^[^ ^3^ ^]^

A deeper understanding of molecular and cellular mechanisms is particularly important in the area of bone regeneration and fracture healing. Bone diseases, such as osteoporosis, osteoarthritis, and bone cancers, impact bone density, structure, and function, often leading to fractures, pain, and mobility issues.^[^ ^4^ ^]^ In research, these conditions are studied using advanced imaging techniques, in vitro cell models, and in vivo animal models to analyze bone remodeling, mineral density, and cellular interactions. Cutting‐edge technologies, including 3D bioprinting and omics approaches, further enable the simulation of disease environments and the development of targeted therapies.^[^ ^5^ ^]^ However, research on bone diseases faces significant challenges, particularly in replicating the complex microarchitecture and dynamic remodeling of human bone as well as its unique biomechanical environment in vitro.^[^ ^6^, ^7^ ^]^ Although animal models remain indispensable for investigating long‐term and systemic aspects of bone physiology, they have substantial limitations in mimicking initial processes of human bone development and fracture healing. These limitations are not only mechanical in nature, e.g., related to species‐specific differences in load‐bearing and regeneration, but also biological, including different bone metabolism rates and signaling sensitivities (e.g., bone morphogentic protein thresholds). Our platform aims to overcome these challenges by enabling the controlled investigation of early cellular and molecular processes under defined human‐relevant mechanical and biochemical conditions. This approach enables the targeted investigation of human‐specific cellular responses to biomechanical cues, which cannot be isolated from systemic factors in conventional animal models.^[^ ^8^, ^9^ ^]^

Generally, one of the main objectives in tissue engineering is to develop systems that allow for precise regulation of cell culture conditions. Advanced bioreactor systems, microfluidic devices, and mechanical stimulation methods are increasingly used to closely monitor and adjust environmental factors such as temperature, pH, oxygen saturation, nutrient supply, and mechanical loading. These setups allow for a constant supply of fresh nutrients to cell cultures, tissues, and composite tissues while providing permanent control over experimental conditions. Moreover, they allow for accurate regulation of medium flow and seamless integration with other systems, improving the modeling of cell‐cell and cell‐environment interactions.^[^ ^10^, ^11^ ^]^

Another important aspect in tissue engineering is the simulation of mechanical stresses, which play an essential role in tissue maturation and growth under physiological conditions. However, when altered in a pathophysiological context, they also contribute to developing disorders such as rheumatoid arthritis, osteoarthritis, and osteoporosis.^[^ ^12^, ^13^, ^14^ ^]^ Mechanical stimulation methods such as pressure and stretch applications are necessary to promote cell behavior and tissue development, particularly in applications such as bone regeneration.^[^ ^15^, ^16^ ^]^

The continuous improvement and integration of these advanced technologies into modular and flexible systems represents a major step forward in the production of functional tissues in vitro. Such systems not only enable more precise control of culture conditions but also improve reproducibility and scalability. These advances open up new opportunities in clinical research and the development of personalized medicine, potentially helping to develop tailored therapies for patients. The targeted simulation of physiological conditions in tissue engineering systems could also help to develop better diagnostic models, which could be of great importance in researching diseases such as cancer, rheumatic diseases, or bone disorders such as osteoporosis.

Building on these advancements, the underlying study aimed to develop a system capable of simulating bone homeostasis and diseases over extended periods, incorporating mechanical load simulation. This system is designed to provide insights into both healthy bone function and pathological conditions. We evaluated this system using the example of bone regeneration following fractures, drawing on our previous experience.^[^ ^17^, ^18^, ^19^, ^20^ ^]^ This approach allows us to study bone healing in a controlled and dynamic environment, reflecting both biological processes and mechanical stress. Therefore, our system is not intended as a complete replacement for animal models, but rather as a complementary platform that can specifically model human cellular processes in a controlled, mechanistically relevant context. Unlike classical 2D or static 3D cultures, our platform allows the observation of differentiated human cells under physiologically relevant mechanical stimulation.

## 2 Results

The study aimed to develop a microphysiological system that simulates bone homeostasis and disease progression over extended periods, including mechanical load simulation. The system we set up here shall provide insights into normal bone function and pathological conditions. We tested it using a bone fracture regeneration model by incubation 3D in vitro fracture hematoma (FH) models, building on our previous work and the limitations we experienced.^[^ ^19^, ^20^ ^]^ This approach lets us study bone healing in a controlled, dynamic environment that reflects bone regeneration processes. In addition, we implemented a mechanical loading unit to simulate the effect of weight bearing on bone healing and homeostasis.

### 2.1 The Modular Bioreactor Platform Enables Precise Control and Dynamic Perfusion of Tissue Models Under Biocompatible Conditions

In the first approach, we developed a bioreactor platform equipped with a microphysiological system for controlling media flows, sensors to standardize and monitor the incubation of monolayer cultures, 3D models, and tissue explants.

The monitoring of our bioreactor setup is ensured by an intuitive and easy‐to‐use web application and enables precise regulation of various parameters essential for maintaining optimal cell culture conditions. The system allows web‐based control, monitoring, recording, and adjustment of important parameters from any location. These include the pH controller, medium flow rates, and temperature controller. The functionality of the system can also be checked at any time via the web app (Figure S1, Supporting Information). Settings for medium flow rates ensure, on the one hand, a controlled and stable nutrient supply while facilitating waste removal, which is crucial for sustaining long‐term cell viability and function. On the other hand, the systems allow for implementing various shear stresses through adjusting the perfusion rate, thereby facilitating both physiological and pathophysiological setups (Figure S1, Supporting Information).

**Figure 1:**
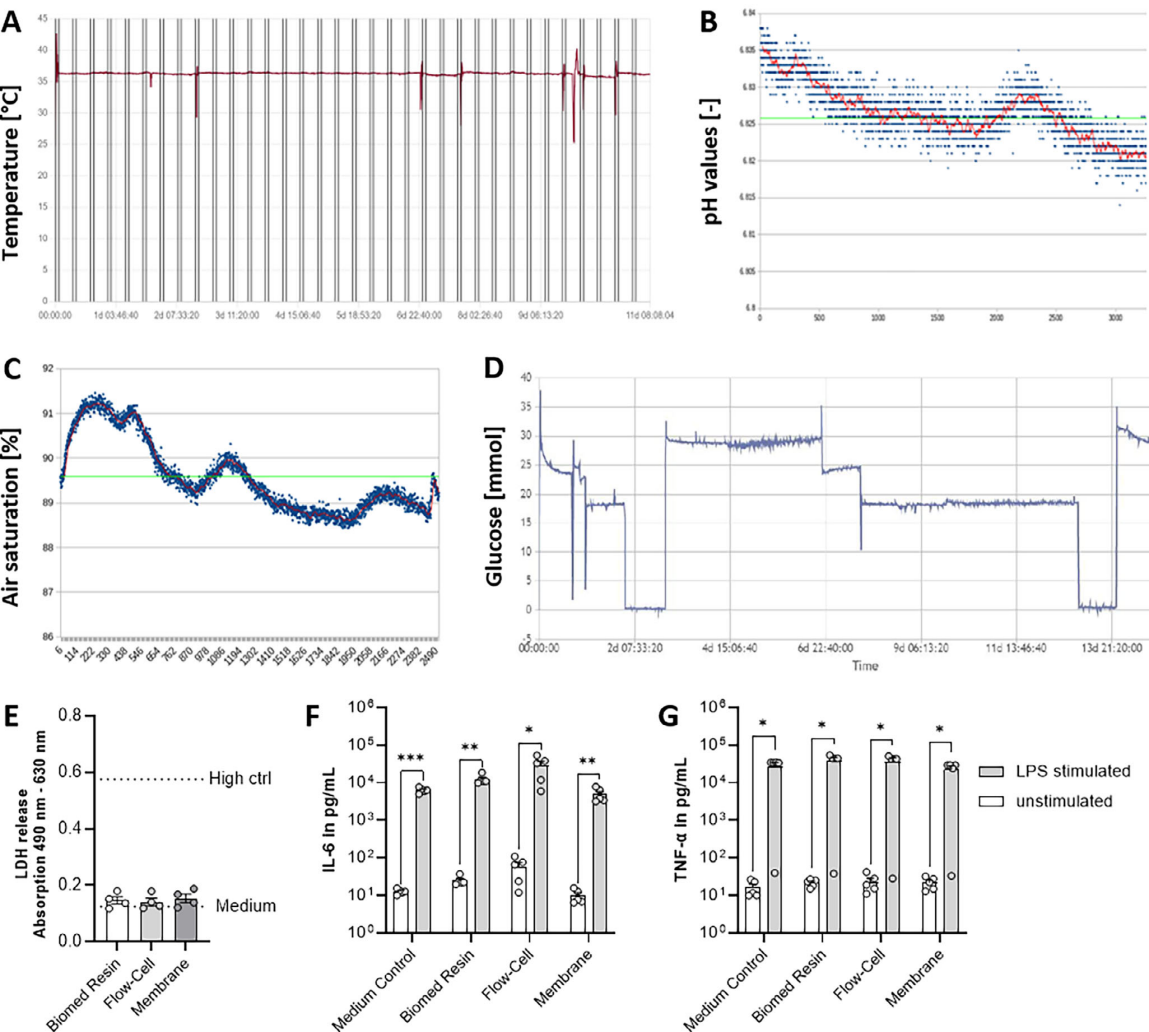
Graphical evaluations of measured values such as temperature, pH value, oxygen saturation, and the glucose sensor and analysis of biocompatibility. A) shows the course of the temperature in the closed system over time. B, C) display an exemplary pH value curve and the exemplary dissolved oxygen concentration over time, which can be analyzed directly in the system and adjusted if necessary. D) shows the calibration and functional testing of the glucose sensor. E) Materials used in the microfluidic cultivation chamber directly in contact with cells/tissues during cultivation do not show cytotoxic effects as shown by LDH release. Graphs are shown as bar charts and individual values with scanning electron microscopy (SEM (*n* = 4)). The upper dotted line represents the high control using Triton X100, while the lower one represents the medium control. F, G) In vitro material testing showed no detectable IL‐6 or TNF‐α release using the whole blood assay, indicating a lack of proinflammatory response of the tested material. Graphs are shown as bar charts and individual values with SEM (*n* = 4). Statistical analysis was performed using 2‐way ANOVA. P‐values are indicated in the graphs with *p < 0.05, **p < 0.01, ***p < 0.001.

The precise monitoring of the temperature is also of utmost importance. A heating plate ensures the cell area stays at the desired temperature, and the bioreactor lid remains. Thereby, the preset is 37 °C, which is the physiological temperature required for optimal cell growth and function. Precise temperature control prevents thermal fluctuations that could compromise cell viability or alter experimental conditions, ensuring reproducibility in long‐term culture experiments (Figure S1, Supporting Information). The bioreactor base station controls the temperature with heating elements and fans. Sensors monitor the temperature, and the software adjusts heating to maintain 37 °C (**Figure** **1**A). Additionally, the system allows for setting a desired pH value by the controlled addition of air and CO_2_ via adjustable flow rates (air flow target and CO_2_ flow target). Maintaining a stable pH is essential for cellular homeostasis, as fluctuations can impact cell metabolism, proliferation, and differentiation (Figure 1B; Figure S1, Supporting Information). The pH value is measured in each medium circuit using an optical sensor from Pyroscience. A software‐based controller adjusts the pH value to the specified target value based on the provided pH values of the medium by regulating the ratio of air to CO_2_ to ensure physiological conditions for the cell cultures. While increasing the CO_2_ infusion lowers the pH value of the medium, reducing the CO_2_ infusion increases the pH level. The same approach is applied to dissolved oxygen levels by regulating the application of compressed air (Figure 1C). Additionally, a glucose sensor integrated into the system also allows monitoring of the glucose concentration over time in order to draw conclusions about the metabolic activity of cultivated cell systems (Figure 1D).

These easy‐to‐use, intuitive implementations with integrated medium flow, pH, and temperature control are essential for creating a stable and physiologically relevant microenvironment for in vitro cell culture.Controlled medium perfusion mimics natural nutrient exchange, pH regulation ensures optimal biochemical conditions, and precise temperature maintenance supports cellular function and differentiation. Together, these factors contribute to higher experimental reproducibility, improved cell viability, and the ability to culture sensitive cell types or engineered tissues over extended periods. This is particularly important in applications such as tissue engineering, drug testing, and disease modeling, where precise environmental control is critical for reliable results.

This sophisticated system ensures an optimal environment for cell cultures by precisely controlling conditions. Its modular design adapts to various experimental needs, ensuring standardized procedures and optimal results.

Next, we evaluated material biocompatibility using an lactate dehydrogenase (LDH) assay. Cells in contact with the system showed LDH levels similar to standard plates and well below the Triton X‐100 cytotoxic threshold, supporting the materials’ suitability for in vitro applications (Figure 1E). We then evaluated the immunogenicity of the microfluidic materials using an in vitro whole blood assay that measured interleukin (IL)‐6 and tumor necrosis factor (TNF)‐α release (Figure 1F,G). The materials did not induce detectable cytokine levels, with responses similar to the negative control (medium only), while lipopolysaccharide (LPS; positive control) triggered a significant inflammatory response.

These results demonstrate that the microfluidic system does not provoke a cytotoxic or an immune response, supporting its suitability for long‐term cell culture and tissue engineering applications.

### 2.2 Perfusion Leads to a Long‐Lasting Survival and Triggered Osteogenesis in an In Vitro FH Model

**Figure 2:**
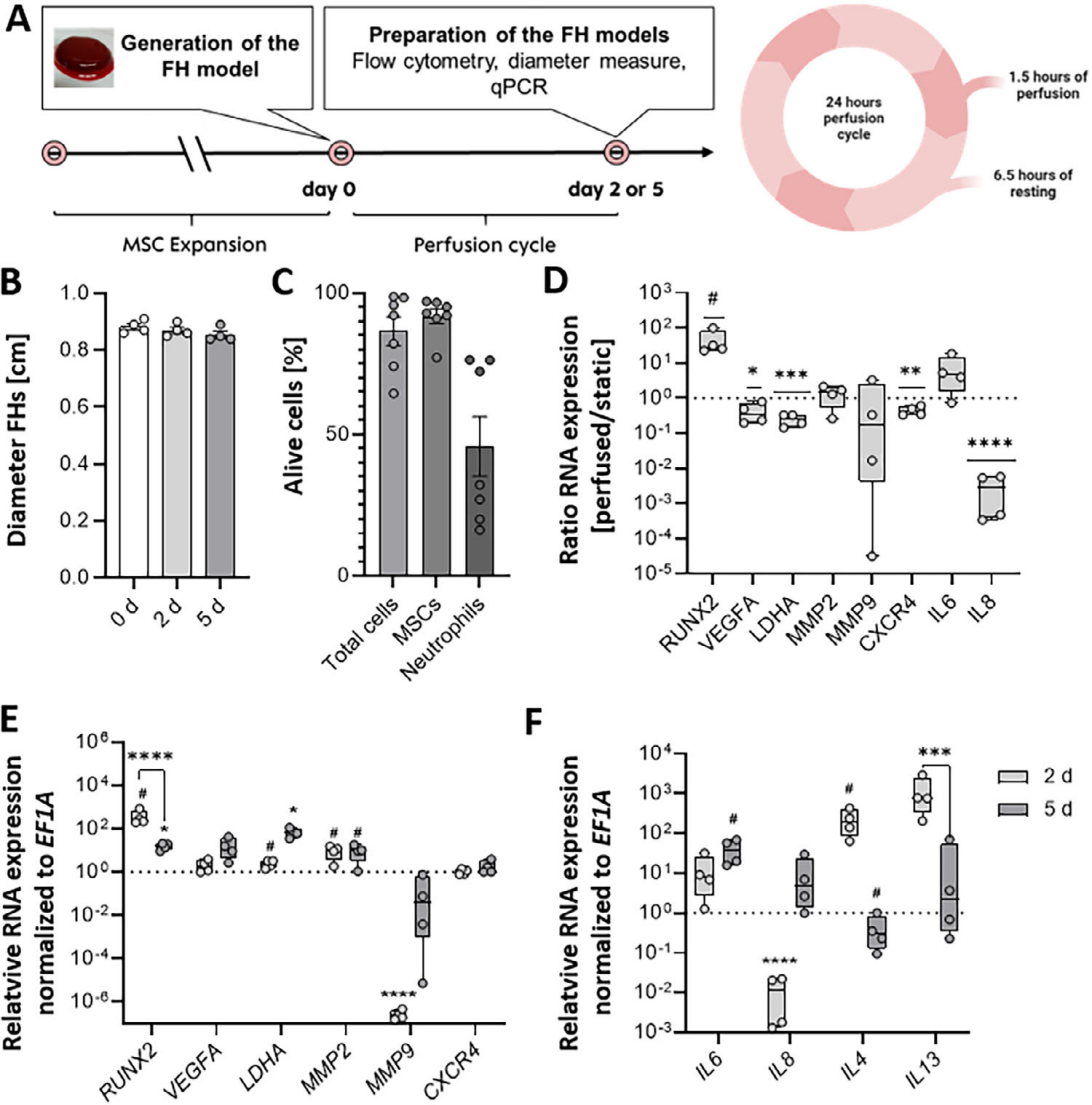
Perfusion improved the integrity and cell viability of the FH model and resulted in an RNA pattern representing the initial phase of fracture healing. A) Schematic illustration of the cultivation profile B) Diameter of the in vitro FHs before and after incubation (*n* = 4) and C) the frequency of total cells, MSCs (CD73^+^, CD90^+^, and CD45^−^), and neutrophil granulocytes (CD45^+^, CD15^+^), negative for 7‐AAD in the in vitro FHs cultured in osteogenic differentiation medium for 5 days (*n* = 7). Data are shown as bar charts with SEM. Statistics were performed using the Mann‐Whitney U test. D) Relative gene expression of osteogenic (RUNX2), angiogenic (VEGFA), metabolic (LDHA), matrix‐degrading and migratory (MMP2, MMP9, CXCR4), and inflammatory (IL6, IL8) marker genes after incubation of the in vitro FH models in osteogenic medium for 2 days under perfused conditions, compared to static conditions (dotted line) for *n* = 4. Data are shown as box plots. Statistical analysis was performed using the one‐sample t‐test versus the hypothetical value 1. The dotted line represents the hypothetical value 1 under static conditions. *P*‐values are indicated in the graphs with #*p* > 0.1, *p < 0.05, **p < 0.01, ***p < 0.001 ****p < 0.0001. E) Relative gene expression of osteogenic (RUNX2), angiogenic (VEGFA), metabolic (LDHA) matrix‐degrading and migratory (MMP2, MMP9, CXCR4) and F) inflammatory (IL6, IL8) and anti‐inflammatory (IL4, IL13) marker genes after incubation of the in vitro FH models in osteogenic medium for 2 or 5 days under perfused conditions (*n* = 3‐4) compared to day 0 (dotted line). Data are shown as box plots. Statistical analysis was performed using the one‐sample t‐test to compare 2 and 5 days with 0 days, and the t‐test to compare 2 and 5 days. P‐values are indicated in the graphs with #*p* < 0.1, *p < 0.05, ***p < 0.001, ****p < 0.0001.

Using our designed bioreactor platform with the integrated microfluidic cultivation chamber (Figures 6 and 7) and the experimental design described in **Figure** **2**A, we could extend the cultivation period of the 3D in vitro FH models – bone fracture regeneration models.

Extending the incubation period of the FH models from two days under incubation conditions without constant medium perfusion to up to five days using perfusion conditions did not alter the morphology and diameter of the models (Figure 2B). Flow cytometry analysis revealed an average of 76% living 7‐AAD^−^ total cells after 5 days of cultivation. While MSCs (CD73^+^, CD90^+^, and CD45^−^) survived the cultivation at a percentage of slightly below 90%, ≈48% of neutrophil granulocytes (CD45^+^, CD15^+^) survived (Figure 2C).

We concluded that the perfused system has significant advantages over static conditions, leading to prolonged cell survival. Perfusion did not notably affect MSC survival, which is crucial in early fracture healing.

The next step was to find out how perfusion affects gene expression in the in vitro FH models. Therefore, we compared the RNA expression pattern after 2 days of incubation in the osteogenic medium under either perfused or static conditions (Figure 2D). For static conditions, the in vitro FH models were incubated for 2 days in a 48‐well plate as reported earlier.^[^ ^20^ ^]^

Comparing these data, we observed a trend of higher expression of the osteogenic transcription factor *runt‐related transcription factor 2* *RUNX2* under perfused conditions. In contrast, the angiogenic marker *vascular endothelial growth factor A* (*VEGFA*) was significantly lower expressed under perfused conditions than in the static control. *Matrix metalloproteinase* (*MMP*)2, *MMP9*, and *IL6* showed no differences between the two cultivation methods. However, *C‐X‐C chemokine receptor type 4* (*CXCR4*), *IL8*, and *lactate dehydrogenase A* (*LDHA*) were significantly lower expressed when perfusion was applied (Figure 2E). Interestingly, *IL4* and *IL13* were not differentially expressed under static but under perfused conditions (Figure 2D,F).

Next, we analyzed gene expression changes in the FH model over an incubation period of up to five days. The osteogenic marker *RUNX2* was significantly upregulated at days 2 and 5 compared to baseline, though expression declined at day 5 relative to day 2. *VEGFA*, an angiogenesis marker, showed a numerical increase over time. *LDHA* expression trended upward at day 2 and reached statistical significance by day 5. *MMP2* expression was elevated at both time points, with no difference between days 2 and 5, whereas *MMP9* was significantly reduced at day 2 but returned to baseline by day 5. The migration marker *CXCR4* exhibited only a slight increase (Figure 2E). *IL6* expression rose modestly at day 2 and further at day 5. The inflammatory marker *IL8* was significantly downregulated at day 2 but slightly increased at day 5 relative to both baseline and day 2. Interestingly, the anti‐inflammatory markers *IL4* and *IL13* were significantly elevated at day 2 but downregulated at day 5 compared to their earlier peak (Figure 2F).

Our findings demonstrate that our approach effectively extends the incubation period for FH models from 2 to 5 days. This extension will allow for a more comprehensive observation of fracture healing, capturing both the early stages and the transition through different phases of bone repair. The modular expansion of the bioreactor platform allows precise application and monitoring of pressure.

### 2.3 Modular Expansion of the Bioreactor Platform Allows Precise Application and Monitoring of Mechanical Loading

**Figure 3:**
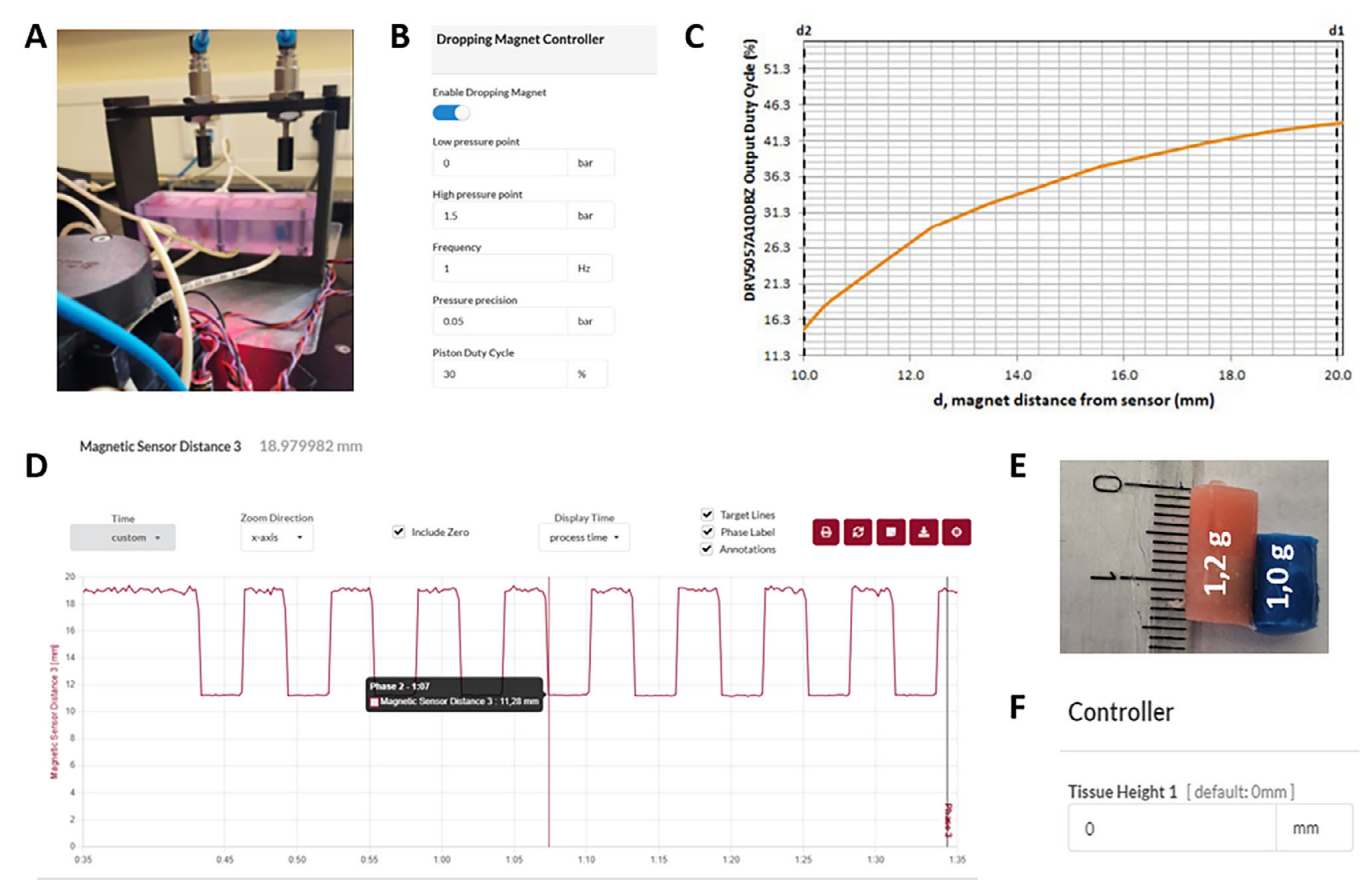
Validation of tissue constructs subjected to mechanical compression. A) An experimental setup is used to validate the system under real conditions with connected control and supply lines. B) The user interface for controlling the pressure application unit with adjustable parameters such as pressure, frequency, and duty cycle. C) Calibration curve of the magnetic sensor for recording the movement amplitude as a function of the distance. D) Data output of the control software for analyzing the real‐time signal curves of the pressure application unit. This allows the diameter of integrated tissue models to be monitored. E) Exemplary illustration of two silicone gaskets with integrated magnets for loading tissue models. F) Interface to set the starting diameter of the implemented tissue.

Testing the application and monitoring of mechanical loading under real laboratory conditions (**Figure** **3**A), we demonstrated reliable actuator control via a custom user interface. The system allows independent control of each pressure module, with pressure adjustable from 0 to 1.5 bar and frequencies ranging from 0.1 to 10 Hz in 0.1 Hz increments via a web application. The unit uses a pneumatically controlled valve to raise and lower a magnet that, in turn, moves a silicone piston inside the microfluidic cultivation chamber without direct contact. By adjusting the frequency of the piston's movement, the tissue or organoid is periodically compressed, with the piston's weight determining the load intensity (Figure 3B). Additionally, silicone gaskets of varying sizes and weights enable the generation of different pressure profiles tailored to specific experimental needs (Figure 3E). The system continuously monitors the magnet—and therefore the piston—position using a Hall effect sensor placed beneath the microfluidic cultivation chamber. This sensor records the magnetic field strength, enabling accurate calculation of the piston's position (Figure 3C). A second magnet, mounted on a pneumatic cylinder above the microfluidic cultivation chamber, further refines the control of the silicone piston. Signal curves from the control software confirm consistent and reproducible activation of the system (Figure 3D).

Moreover, the system can record changes in the tissue models' size over time (with an adjustable starting diameter,Figure 3F) to assess morphological changes resulting from mechanical stimulation. Developed independently of the bioreactor platform, this pressure application unit is designed for flexible placement and can be easily integrated or removed from the microfluidic cultivation chamber based on experimental requirements.

In summary, the developed microfluidic system delivers highly precise and controllable mechanical stimulation. By integrating pneumatic compression, magnetic control, and sensor‐based feedback, this innovative platform opens up diverse possibilities for advancing tissue engineering and cell mechanobiology research.

### 2.4 Application of Defined Mechanical Loading Conditions Led to Tissue Models with Bone‐Like Properties

**Figure 4:**
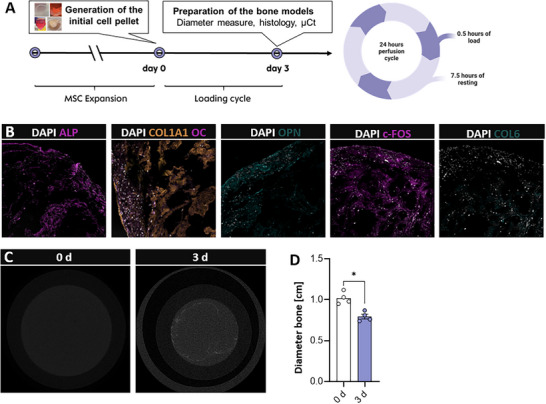
Immunofluorescence staining confirms increased expression of osteogenic markers, highlighting the potential of the system to simulate in vivo‐like mechanical environments for tissue engineering applications. A) Schematic illustration of the cultivation profile B) Illustrates the immunofluorescence staining of alkaline phosphatase, collagen type 1A1 and osteocalcin, osteopontin, c‐FOS, and collagen type 6. To reveal the nuclei of present cells, all slides were counterstained with DAPI (*n* = 4). All scale bars = 50 µm. Images are exemplary C) Reconstruction of µCT. Exemplary image of *n* = 3. D) shows the diameter of the bone models before and after mechanical loading for 3 days (*n* = 4).

To evaluate the compression unit's functionality, we employed our mesenchymal condensation‐based MSC model to generate standardized bone models. Following initial generation (Figure 8), the constructs were cultured, mechanically loaded (**Figure** **4**A), and subsequently analyzed for bone‐specific protein expression via immunofluorescence staining. DAPI staining confirmed a homogeneous cell distribution, though a higher cell density was noted along the construct borders, forming a distinct lining layer (Figure 4B). Immunostaining revealed osteopontin —primarily produced by pre‐osteoblasts and immature bone cells— and osteocalcin, which is predominantly expressed by mature osteoblasts. This suggests the presence of cells at various differentiation stages within the constructs. In addition to these non‐collagenous proteins, we observed marked expression of collagen I and collagen VI, proteins typical of bone extracellular matrix. Alkaline phosphatase, a key marker of osteogenic differentiation, was distinctly expressed, alongside c‐FOS, a protein critical for osteogenic differentiation and indicative of cellular adaptation to mechanical loading (Figure 4B). Micro‐computed tomography (µCT) analysis confirmed early mineralization of the bone constructs (Figure 4C). Additionally, our bioreactor enabled continuous monitoring of tissue diameter over time by internally measuring the distance of a magnet from the reading unit. This analysis revealed that the construct diameter decreased by ≈30% after three days of compression, indicating a substantial increase in tissue density (Figure 4D).

In our setup, we used a silicone gasket weighing 1.2 g (Figure 3E). The force exerted on the tissue was calculated using F = m×g, yielding 0.0118 N. Given an average bone model diameter of 1 cm (r = 0.5 cm), the contact area was computed as A = π × r^2^, resulting in 7.85 × 10^−8^ m^2^. Accordingly, the mechanical pressure is σ = 0.0118 N / (7.85 × 10^−8^ m^2^) ≈ 150.3 kPa, which closely approximates the loading pressure in human cartilage.

Our developed setup successfully generated standardized bone models and reliably measured relevant pressure parameters, enabling the evaluation of key tissue properties. Therefore, the system offers the possibility of determining plastic compression properties of the integrated tissue or scaffold.

### 2.5 Expansion of the Modular Bioreactor Platform Enables Optimized Co‐Cultivation of Composite Tissue Models

**Figure 5:**
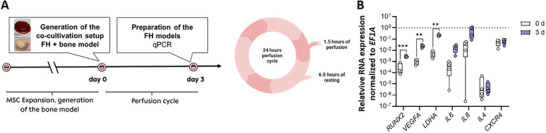
Illustration of the modular design of the cultivation unit, its fluidic path strategy, and the validation of tissue constructs subjected to dual perfusion. A) Schematic illustration of the cultivation profile. In B), the relative gene expression of the osteogenic marker (RUNX2), the angiogenic/metabolic markers (VEGFA, LDHA), CXCR4, reflecting the migratory potential, inflammatory markers IL8 and IL6, and the anti‐inflammatory marker gene IL4 is depicted. Data are shown as box plots with individual data points. For statistical analysis, the results were compared using the t‐test (*n* = 4). P‐values are indicated in the graphs with **p < 0.01, ***p < 0.001.

To assess the functionality of our novel bone constructs and simulate the early phase of fracture healing, we co‐cultivated bone models with FH models under chemically induced hypoxia using deferoxamine (DFO) in a bioreactor for 3 days. In this setup, the upper FH models were perfused with a hypoxic medium containing 250 µmol DFO, while the lower bone models were maintained in standard medium without DFO. Given that our previous studies revealed a significant loss of viable cells in FH models after 3 days, we aimed to determine whether perfused conditions could support prolonged co‐cultivation (**Figure** **5**A).

After 3 days, we analyzed the RNA expression profiles to verify whether a characteristic fracture healing signature was established and to assess the expression of the anti‐inflammatory marker *IL4*, which may indicate the onset of the next healing phase. Our results demonstrated significant upregulation of the osteogenic marker *RUNX2*, the angiogenic marker *VEGFA*, and the metabolic marker *LDHA* compared to baseline (0 h). The pro‐inflammatory marker *IL6* showed an increasing trend, whereas *CXCR4* remained at basal levels. Notably, the inflammatory marker *IL8* was markedly elevated, and *IL4* exhibited a numerical increase, suggesting the initiation of an anti‐inflammatory shift (Figure 5B).

These findings confirm that our bone constructs can trigger characteristic processes of the fracture hematoma without the need for osteogenic induction media. In conclusion, our innovative setup allows us to i) produce standardized bone constructs exhibiting typical bone marker expression and early mineralization and ii) precisely cultivate different tissues in a co‐culture configuration, thereby providing a robust platform for studying early fracture healing mechanisms.

## 3 Discussion

To generate a valid method for incubating macroscale tissue models under the most native conditions possible, we developed a perfusion platform with a printed inert microfluidic cultivation chamber that allows the perfusion of composite model systems. In addition, we have developed a mechanical compression device that can be attached to the perfusion platform based on a modular principle. Using this system, we demonstrate that perfusion alone enabled a significant prolongation of the incubation period of our in vitro model of the initial phase of fracture healing, which we established in a previous study. Furthermore, applying this system allowed us to successfully mimic —apart from the initial inflammatory phase of fracture healing— the transition to the next state, the anti‐inflammatory phase. By testing the mechanical compression, we could produce standardized, functional bone models with substantial similarities to native bone, as confirmed by immunofluorescence staining and µCT analysis.

To date, it is well recognized that perfused systems offer a promising approach for maintaining cell and tissue culture conditions in particularly organoids and 3D tissue cultures.^[^ ^21^ ^]^ Therefore, the emergence of bioreactors and on‐a‐chip concepts for high‐throughput basic and translational research or drug testing approaches on the macro‐ and microscale has been evident over the last decade.^[^ ^22^ ^]^ Hereby, macroscale in vitro models offer numerous advantages compared to microscale ones. These include greater physiological proximity, better diffusion, natural cell interactions, and the possibility to study systemic effects for *inter alia* representing complex organ interactions.^[^ ^23^ ^]^ However, they are also associated with sophisticated technical requirements, increasing costs, high complexity, and low flexibility. Because compromises must be made, conventional bioreactors often struggle to provide the necessary experimental needs and flexibility. These systems maintain stable pH, constant oxygen supply, or nutrient gradients; however, often without real‐time monitoring and automated control.^[^ ^24^ ^]^ The bioreactor system presented here provides all these features in a modular manner, coming with an intuitive and simple web‐based software for adjusting parameters such as pump speed, temperature, and pH value, providing a suitable tool for long‐term cell culture. This is in line with several studies showing the effect of a controlled microenvironment on cell culture, focusing on cell functionality.^[^ ^25^, ^26^ ^]^ The system is therefore suitable for investigating conditions including hypo‐ or hyperthermia, the influence of shear and compression forces, low and high pH, or different oxygen availabilities on cell culture systems.

The heart of our bioreactor system is the printed inert modular microfluidic cultivation chamber, showing no signs of cytotoxicity and inflammatory responses in our approach (low LDH release, no IL‐6 or TNF‐α induction) and in line with previous reports on the importance of using biocompatible materials for microfluidic tissue culture.^[^ ^27^, ^28^ ^]^

In order to assess the suitability of the bioreactor system for biological studies, we employed an established fracture hematoma model to visualize the initial phase of fracture healing in vitro.^[^ ^20^ ^]^ Previous studies have shown that dynamic culture conditions enhance osteogenesis by improving nutrient transport, waste removal, and mechanotransduction.^[^ ^29^, ^30^ ^]^ Our findings confirm this, as perfusion markedly increased cell viability and the gene expression of the osteogenic marker *RUNX2*, compared to static culture. Interestingly, the observed reduction in *IL8* expression and the increased expression of *IL4* and *IL13* under perfusion indicate a shift toward a pro‐regenerative anti‐inflammatory immune environment, similar to findings in bone fracture healing studies.^[^ ^31^ ^]^

Furthermore, we could show that the expression of *CXCR4*, *VEGFA*, and *LDHA* was significantly lower under perfused conditions compared to static conditions. Under perfusion, cells are continuously supplied with oxygen and nutrients. This could prevent hypoxic conditions and thus reduce the expression of hypoxia‐induced genes such as *LDHA*, *VEGFA*, and *CXCR4*, as it is well known that changes in the microenvironment can trigger these events.^[^ ^32^, ^33^ ^]^

In addition, perfusion‐generated shear forces enhance nutrient delivery and trigger mechanical signals that modulate gene expression. Although research on perfusion's impact on specific genes is limited, current data indicate that dynamic perfusion reduces stress‐related gene expression compared to static culture. This supports the hypothesis that dynamic perfusion can optimize in vitro and ex vivo fracture healing models, potentially advancing personalized therapeutic strategies.

In mechanobiology, it is well established that physical stimuli —whether from the microenvironment or applied externally— critically regulate cell behavior, tissue homeostasis, development, and differentiation.^[^ ^34^ ^]^ Imbalances in these stimuli can lead to pathological conditions.^[^ ^35^, ^36^, ^37^ ^]^ To harness these effects, we have integrated a modular mechanical loading unit into our bioreactor system, overcoming limitations seen in conventional setups.^[^ ^38^ ^]^

Traditional bioreactor systems, such as fed‐batch or spinner‐flask models, generate turbulent shear forces that can damage sensitive cells and lack precise control over mechanical stimulation.^[^ ^39^ ^]^ Rotating wall vessels offer a dynamic environment; however, their fluid dynamics are difficult to manage, often resulting in uneven nutrient distribution and indirect mechanical cues.^[^ ^40^ ^]^ While perfusion bioreactors provide continuous nutrient flow, they generally limit mechanical input to fluid shear and require complex modifications for targeted stress application.^[^ ^41^ ^]^ Microfluidic systems, though excellent for precise control, are constrained by small culture volumes, making them unsuitable for mechanically stressed tissues like bone.^[^ ^42^ ^]^

Our modular design addresses these challenges by enabling simultaneous, precisely controlled mechanical loading across multiple samples. Digital control over parameters such as pressure, frequency, and piston position ensures targeted mechanical stimuli, thereby enhancing reproducibility and scalability. Integrated sensor technology permits real‐time monitoring and adjustment of mechanical parameters, making the system ideal for preclinical research in tissue regeneration.

Bone tissue, in particular, is highly responsive to mechanical cues. In vitro, mechanical stimulation promotes osteogenic differentiation of mesenchymal stromal cells, as evidenced by increased alkaline phosphatase activity, collagen type I synthesis, and upregulation of osteogenic markers such as RUNX2.^[^ ^43^, ^44^ ^]^

To test our bioreactor's effectiveness in generating bone models from mesenchymal stromal cell (MSC) pellets, we applied a cyclic loading regime at 1 Hz for 30 min, followed by a 7.5‐hour rest, over three days. This regimen was selected based on evidence that dynamic, cyclical loading with rest intervals more effectively induces osteogenesis than static loading.^[^ ^45^ ^]^ Bone cells —foremost during initial bone development— are most responsive at frequencies between 1 and 10 Hz, with 1 Hz reliably triggering osteogenic responses.^[^ ^46^, ^47^ ^]^ Our loading protocol resulted in bone constructs with clear osteogenic protein expression and early mineralization, as confirmed by immunofluorescence staining and µCT analysis. The system's digital control, accessible via a web application, allows for easy adjustments of loading parameters, facilitating the optimization of stimulation regimes for standardized bone model generation. Furthermore, our system enables the simulation of both underload and overload conditions by adjusting loading frequency and force, allowing for a broader exploration of mechanobiological responses and the testing of diverse biomaterial types under controlled, dynamic conditions. Since the mechanical environment changes dynamically during bone healing and higher compressive and tensile loads occur, especially in later phases, our system enables a phase‐dependent simulation of these mechanical changes by gradually adjusting load and frequency. However, our model has, for now, important limitations: For example, multiaxial stressors, which play a crucial role in cell function and differentiation in vivo,^[^ ^16^ ^]^ cannot yet be adequately modeled. These limitations currently limit the complete reproducibility of the physiological conditions of bone healing. Nevertheless, our platform offers a valuable approach for investigating, in particular, the early phases of bone regeneration under human‐relevant mechanical and biochemical conditions, thus enabling an important step toward validly predicting biological responses to biomaterial‐assisted therapies.

In order to broaden the system's applicability, we have developed an integrated microfluidic cultivation chamber for composite tissue models requiring dual medium supply. This chamber features a second fluid pathway, enabling simultaneous, distinct perfusion for different tissue types. In validation studies using a fracture gap model^[^ ^19^ ^]^ in which an FH model was co‐cultured with a bone model for three days, the bone compartment received osteogenic medium while the adjacent compartment was perfused with a hypoxia‐mimicking medium supplemented with 250 µmol DFO. This dual perfusion approach enables precise control over nutrient and mechanical stimuli, facilitating the study of complex interactions among cells, growth factors, and mechanical forces.

Our results showed that the bone model induced osteogenic, angiogenic, and inflammatory responses in the co‐cultured tissue, with significantly elevated expression of *RUNX2*, *VEGFA*, and *IL6* after 3 days. These findings confirm the osteoinductive capacity and functional integration of our bone models. Future applications include developing composite models incorporating tissues essential for joint homeostasis —such as bone, bone marrow, tendons, and muscle— to accurately simulate physiological conditions for long‐term tissue function, drug testing, and biomechanical studies in musculoskeletal research. The successful use of our system has shown that the combination of precise mechanical stimulation and a controlled microenvironment enables differentiated investigation of cellular responses that often cannot be adequately captured in conventional animal models or classic in vitro systems.

## 4 Conclusion

Here, we developed a novel platform to incubate macroscale tissue models under near‐native conditions, integrating a modular mechanical compression device. This system not only extends the viable incubation period of an in vitro fracture healing model by transitioning it from an inflammatory to an anti‐inflammatory phase but also produces standardized, functional bone models that closely resemble native bone. The platform, equipped with a user‐friendly web interface for real‐time control of parameters such as pump speed, temperature, and pH, improves nutrient supply and mechanotransduction compared to static culture. Additionally, the dual perfusion approach enables composite tissue models to receive tailored media supplies, facilitating studies on tissue interactions and healing processes. Thus, we conclude that our modular bioreactor system represents a significant advancement in in vitro tissue modeling by combining dynamic perfusion with precise mechanical stimulation. Its ability to maintain a controlled microenvironment and simulate complex physiological conditions makes it a promising tool for preclinical research, drug testing, and the investigation of tissue regeneration mechanisms.

## 5 Experimental Section

### Setup of the Novel Modular Bioreactor Platform

**Figure 6:**
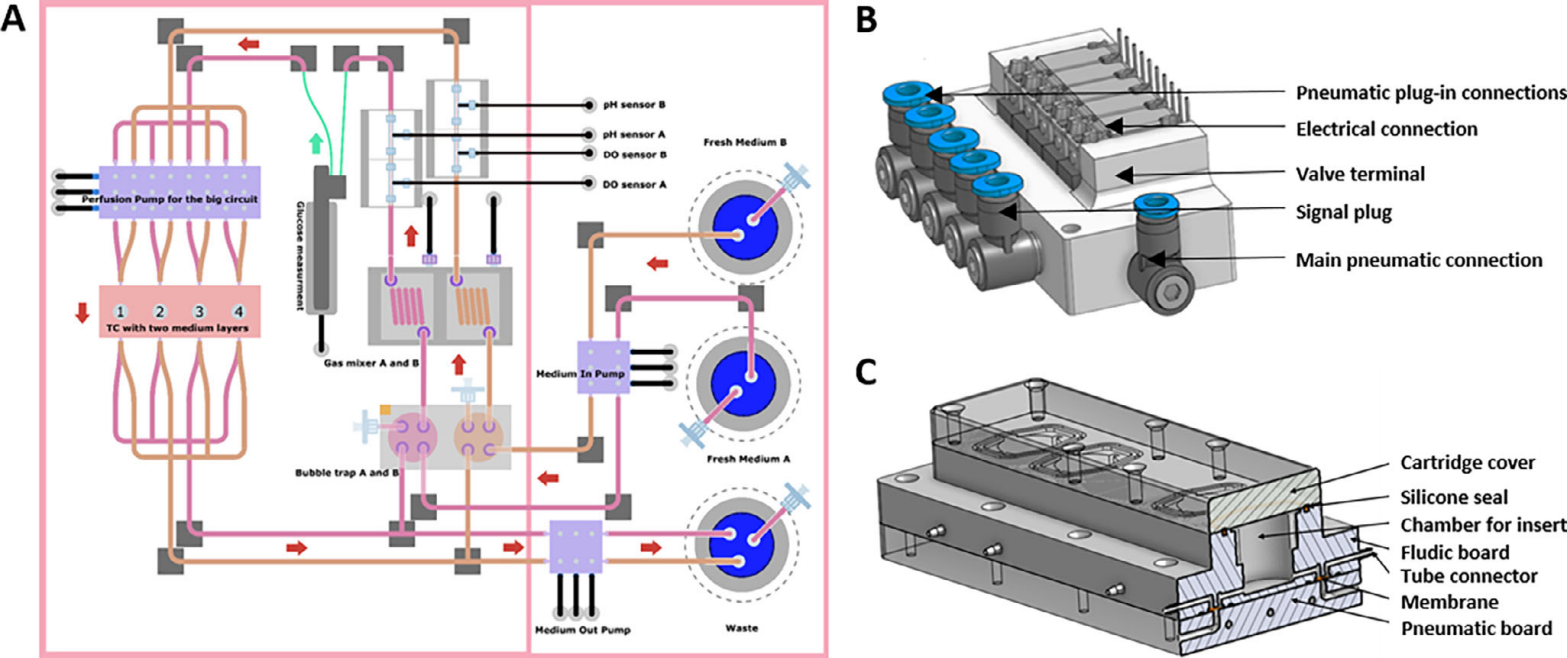
Schematic representation of our modular bioreactor setup and the integration of the microfluidic cultivation chamber. A) shows the central perfusion circuit, which ensures a continuous supply of fresh medium to the cell cultures. B) shows the pneumatic valve terminal that serves as a central control element. The blue caps indicate pneumatic connections, while several valve units are integrated in a common housing. Electrical connections are located on the top, which are used for the targeted control of the valves. Lateral air connections enable precise control of compressed air, allowing the valve terminal to ensure efficient control in the automated process. C) 3D representation of the architecture of the insert‐based microfluidic cultivation chamber based on Biomed Resin. It consists of four culture compartments, channels, and connections that enable precise control of the medium flows. Several inlet and outlet connections for the pneumatic pump system ensure easy integration into the perfusion circuit of the bioreactor setup. The inner diameter of the tubing used for perfusing the microfluidic chamber is 750 µm.

The bioreactor platform owns two gas inlets, one for compressed air and one for CO_2_. In addition, it comes with two media reservoirs and one medium waste tank –each equipped with a peristaltic pump– to provide two different types of media in two separate central perfusion circuits. The central perfusion circuits equipped with space‐saving quake pumps ensure a continuous supply of fresh medium to the tissue culture unit on a heating platform in the system's center. Two gas mixers ensure the dissolved gas management, regulating the composition of oxygen provided with compressed air and carbon dioxide in the medium circuit. A silicone gas exchanger allows a gas mixture of air and CO_2_ to diffuse into the medium. Bubble traps remove undissolved air bubbles to keep the system's sensors in the fluidic path functional (**Figure** **6**A,B). For perfused long‐term cell and tissue culture, an insert‐based microfluidic cultivation chamber using Biomed Resin was designed and fabricated. The 3D architecture of the system (Figure 6C) consists of four independent culture compartments, interconnected by a network of precisely structured microchannels that enable controlled medium perfusion. These microfluidic cultivation chambers have been adapted to be equipped with commercially available cell culture inserts (Corning, USA). Here, inserts that were suitable for a 24‐well cell culture with a membrane pore size of 0.4 µm were used. The system has multiple inlet and outlet ports, allowing seamless integration into the bioreactor perfusion setup. A pneumatically controlled pump system ensures stable and continuous media flow, crucial for maintaining a physiologically relevant microenvironment.

### Modular Design of the Cultivation Units Including Mechanical Components for the Application of Mechanical Load

**Figure 7:**
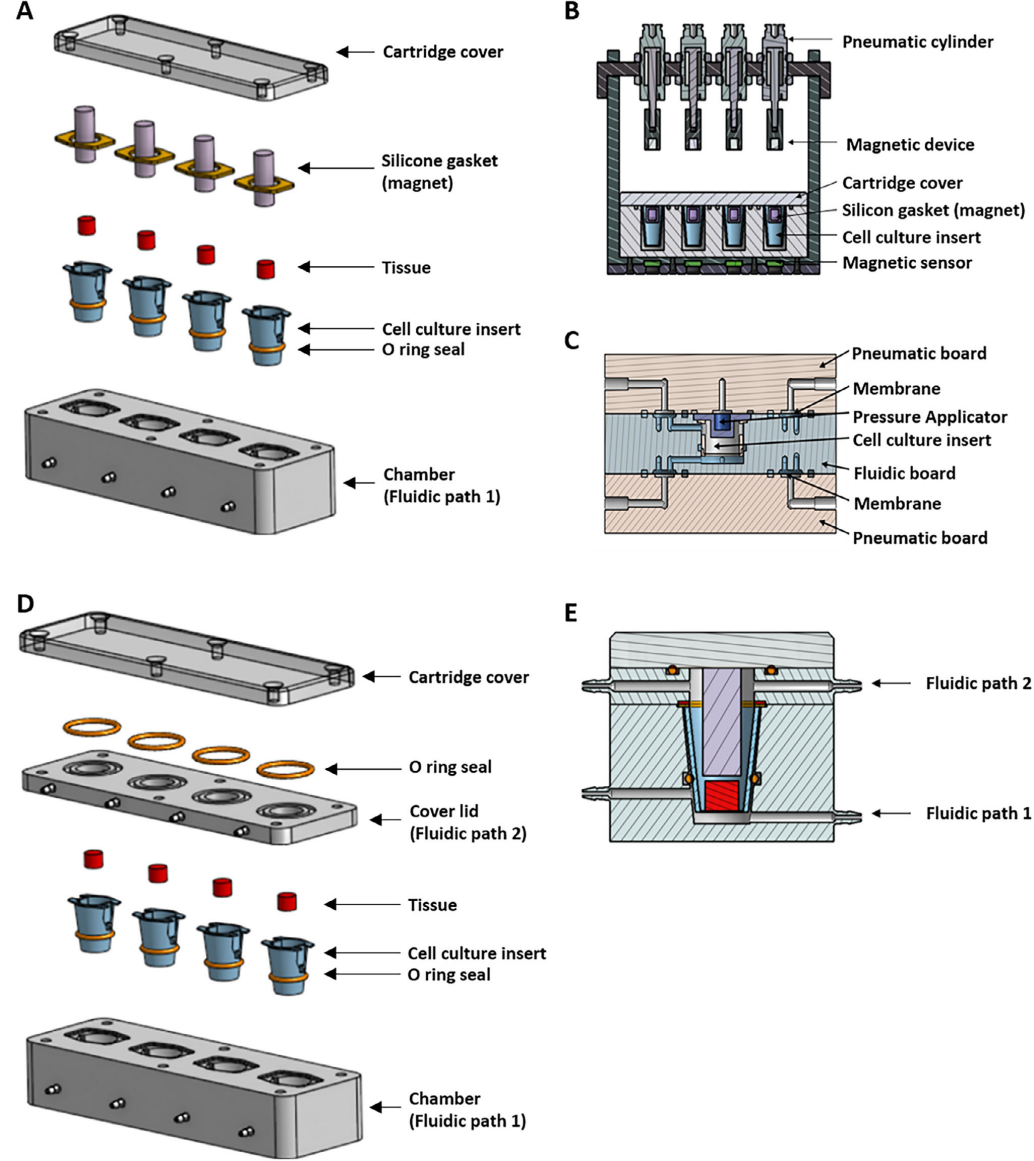
Illustration of the modular design of the cultivation unit, its mechanical components. A) Exploded view of the components of the newly designed microfluidic cultivation chamber, illustrating the detailed assembly. The parts include (from top to bottom): the top cover with screw holes, O‐ring seals for a tight closure, the silicone gaskets with integrated magnets, the cell culture inserts used with O‐ring seals, and the bottom housing for the fluid path. The design enables a modular structure for up to 4 parallel microfluidic cultivation chambers B) shows the cross‐section of the pressure application unit, which was specially developed for processing several samples in individual chambers. The upper section shows the pneumatically controlled cylinders for the application of mechanical pressure, which are controlled by precise mechanics. Each cylinder module works synchronously to maximize efficiency and minimize sources of error. C) represents a cross‐sectional view of the pressure application unit integrated in the cultivation unit. D) Exploded view of the components of the newly designed microfluidic cultivation chamber, illustrating the detailed assembly. The parts include (from top to bottom): the top cover with screw holes, O‐ring seals for a tight closure, a center plate with liquid inlet and outlet channels of the second fluid path, the cell culture inserts used with O‐ring seals, and the bottom housing for the first fluid path. The design enables a modular structure for up to 4 parallel inserts. E) The cross‐sectional view shows the tissue culture chambers with tissue (red) and the silicone gasket (shaded purple). Two different media compositions can flow in and out of the culture chambers to ensure continuous perfusion and supply composite tissue models with the appropriate medium for individual nutrient supply.

The system has been enhanced and optimized to replicate repetitive mechanical loads, such as those experienced during walking. An exploded view (**Figure** **7**A) illustrates its modular design and individual components. A pneumatic compression unit was integrated to enable targeted mechanical force application. Cross‐sectional views (Figure 7B,C) reveal the internal structure of the pneumatically operated actuator, which precisely controls the mechanical stimuli. By modifying the microfluidic cultivation chamber described inFigure 6C to incorporate a second flow path in the coverlid, distinct media to the upper and lower tissues were now delivered (Figure 7D,E). This design enables targeted and independent cultivation of each tissue model with its specifically required medium composition.

### Blood Sampling, Bone Marrow‐Derived MSC Isolation and Cultivation

Preparing in vitro FH models, peripheral blood was collected from healthy donors using EDTA Vacutainers (Becton Dickinson, Franklin Lakes, USA). Next, human MSC were isolated and derived from bone marrow explants of patients undergoing total hip replacement at the Center for Musculoskeletal Surgery, Charité‐Universitätsmedizin Berlin. Explants were distributed via the “Tissue Harvesting” core facility of the Berlin‐Brandenburg Center for Regenerative Therapies. The Charité‐Universitätsmedizin Ethics Committee approved all procedures, which adhered to the Helsinki Declaration (ethical approval EA1/012/13 and EA1/207/17).

To obtain MSC in monolayer culture, the bone marrow was transferred to a 175 cm^2^ cell tissue flask (Greiner Bio‐One, Kremsmünster, Austria). They were incubated in DMEM + GlutaMAX (Gibco, Carlsbad, USA), supplemented with 10% human AB serum (PAN Biontech GmbH, Aidenbach, Germany), 1% Penicillin/Streptomycin (Gibco, Carlsbad, USA), and 20% StemMACS MSC Expansion Media Kit XF (Miltenyi Biotech, Bergisch Gladbach, Germany) in a humidified atmosphere (37 °C, 5% CO_2_). After 2 days, the supernatant was discarded, the adherent cells were washed thrice with PBS, and the cell culture medium was replaced once a week. When the cells reached 80%–90% confluence, they were passaged using Trypsin‐EDTA (Sigma, Munich, Germany). Bone marrow‐derived MSC at passages 3–4 for all in vitro models were used.

### Differentiation and Characterization of Bone Marrow‐Derived MSC

We differentiated human MSC according to established protocols.^[^ ^48^ ^]^ MSC were characterized by immunophenotyping: assessing the expression profile of typical surface markers (CD73^+^, CD90^+^, CD105^+^; CD34^−^, CD45^−^, CD20^−^, CD14^−^, and HLADR^−^) using antibodies provided by Miltenyi (Miltenyi Biotech, Bergisch Gladbach, Germany) according to the manufacturer's instructions. As described in detail in our previous work,^[^ ^20^ ^]^ only cell cultures that met the minimal criteria for MSC set by the Mesenchymal and Tissue Stem Cell Committee of the International Society for Cellular Therapy,^[^ ^49^ ^]^ including differentiation toward adipogenic and osteogenic lineages and expression of the respective surface marker profile, were used for the experiments.

### Quantification of Cytokine Release for Material Testing

The determination of IL‐6 and TNF‐α release after stimulation of human whole blood was performed using a DuoSet ELISA kit (R&D Systems, Minneapolis, USA) according to the manufacturer's instructions. Whole blood stimulation: Freshly collected human whole blood from healthy donors was collected in heparin‐coated tubes. Subsequently, 2 mL of whole blood per well was incubated in a 24‐well plate with the materials to be tested (part of the microfluidic cultivation chamber made of Biomed Resin, part of the screw connection of the flow cell housing made of polysulfone, membrane) with or without LPS, 100 ng mL^−1^ as a positive control. An untreated condition served as a negative control. Incubation was carried out for 24 h at 37 °C and 5% CO_2_ in a cell culture incubator. After incubation, the samples were centrifuged at 1000×g for 10 min, and the supernatant was collected and stored at −80 °C until IL‐6 and TNF‐α analysis.

### Cytotoxicity Assay

Cytotoxicity Detection LDH Kit (Sigma‐Aldrich, Munich, Germany) was performed according to the manufacturer's instructions and as previously described.^[^ ^50^ ^]^ Cell death was induced by 4% (v/v) Triton X‐100 (Sigma‐Aldrich, Munich, Germany) for 24 h (high control).

### Generation of 3D In Vitro Fracture Hematoma Models

The in vitro FH models were generated using an already established protocol.^[^ ^18^, ^20^ ^]^ In brief, 2.5 × 10^5^ hMSCs were centrifuged at 300×g for 3 min at 4 °C in a 96‐well plate (U‐bottom, Greiner Bio One, Kremsmünster, Austria). Subsequently, the cell pellet was resuspended in 100 µL of allogenic EDTA blood at RT. Afterward, 100 µL CaCl_2_ (10 mmol in PBS) was added immediately and incubated for 15 min in a humidified atmosphere (37 °C, 5% CO_2_) to reconstitute the coagulation process leading to the formation of the in vitro FH models for all subsequent experiments.

### Cultivation of 3D In Vitro Fracture Hematoma Models in the Modular Bioreactor Platform

For perfused cultivation, the in vitro FH models were transferred into DMEM with GlutaMAX supplemented with 10% hAB serum, 100 units/ml penicillin, 100 mg mL^−1^ streptomycin, 0.2% β‐glycerophosphate (Sigma Aldrich, St. Louis, USA), 10^−8^ mol dexamethasone (Sigma Aldrich, St. Louis, USA) and 0.002% ascorbic acid (Sigma Aldrich, St. Louis, USA), within this study further referred to as osteogenic medium (OM). FH models were incubated in a humidified atmosphere (37 °C, 5% CO_2_). The incubation was performed for 2 or 5 days in an insert‐based microfluidic cultivation chamber in a modular bioreactor platform.

### Generation of the In Vitro Bone Models

**Figure 8:**
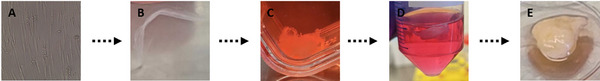
Illustration of the generation of the bone constructs: A) Generation of the initial cell pellet: Overgrown cell layer. B) Detachment of the cell sheet using a cell scraper. C) Initial cell pellet after detachment. D) Cell pellet before incubation for 72 h. E) Cell pellet after 72 h incubation under humidified conditions.

The generation of the in vitro bone models from MSC was based on the preparation and folding of cell sheets, which initially requires overgrown MSC cultures (**Figure** **8**A).^[^ ^17^ ^]^ To this end, confluent MSC monolayers were cultivated for a further 2 weeks with a weekly change of medium. The resulting cell sheets were then washed twice with 32 °C warm PBS, which led to a slight detachment of the cell sheet (Figure 8B). The cell sheet was then carefully detached from the bottom of the 175 cm^2^ cell culture flask to maintain layer integrity using a cell scraper (Figure 8C). The detached cell sheet was centrifuged in fresh medium in a 50 mL tube for 15 min at 250 x *g* at RT, resulting in a folded cell pellet (Figure 8D). The initial cell pellet was incubated in a humidified atmosphere (37 °C, 5% CO_2_) for 3 days, changing the medium daily (Figure 8E).

After generating the initial models, our bioreactor platform was transferred with an implemented system applying mechanical loading. In the experimental design, a frequency of 1 Hz for 0.5 h followed by a resting phase of 7.5 h for 3 days for the mechanical loading was chosen.

### Co‐Cultivation of the FH and Bone Model

After generating the bone models, the FH model and the bone model were placed the FH model on top of each other were combined. The combination of both was transferred into the microfluidic cultivation chamber, within the bioreactor platform, and incubated for 3 days. The FH models were nourished with DMEM with GlutaMAX containing 10% hAB serum, 100 units mL^−1^ penicillin, 100 mg mL^−1^ streptomycin, 250 µmol DFO (inducing hypoxic conditions), while the bone models were nourished with DMEM + GlutaMAX supplemented with 10% hAB serum, 100 units mL^−1^ penicillin, 100 mg mL^−1^ streptomycin. After 3 days, the FH models were separated from the bone models and prepared as shown in the next section.

### Preparation of In Vitro FH Models for Flow Cytometry and Gene Expression Analysis

First, the in vitro FH models were separated from the bone models when co‐culturing was applied for flow cytometry and gene expression analysis. The FH models were washed twice with PBS. Next, the FH models were dissociated by using a cell strainer (70 µm, Corning, New York, USA) to obtain single cells, including red blood cells (RBC), leukocytes, and MSC. RBC lysis was achieved by exploiting their osmotic fragility and incubating the cell mixture with a hypotonic buffer (0.01 mol KHCO_3_, 0.155 mol NH_4_Cl, 0.1 mmol EDTA, pH 7.5) at least twice at 4 °C for 6 min. Subsequently, the remaining leukocytes and hMSCs in PBS with 0.5% BSA (PBS/BSA) were washed and prepared for flow cytometry and qPCR.

### Flow Cytometry Analysis

Cells were washed in PBS/BSA after blocking the nonspecific binding of Fc receptors with a solution containing 5 mg mL^−1^ human IgG (Flebogamma containing IgG_1_ 66.6%, IgG_2_ 28.5%, IgG_3_ 2.7%, IgG_4_ 2.2%, Grifols, Frankfurt, Germany). Then, the cells were stained for 15 min on ice for selected surface markers using anti‐CD45, anti‐CD15, anti‐CD73, and anti‐CD90 with the dilutions in **Table** **1**. The cells were washed with PBS/BSA, centrifuged at 300 x g for 3 min in a U‐bottom 96‐well plate, and the supernatants. The pellets were resuspended in 0.05% NaN_3_ in PBS/BSA. The cells were incubated with 1:100 diluted 7‐AAD (BioLegend, San Diego, USA) for 2 min at RT directly before analysis. Cell assessment was conducted using a BD CANTO II (BD Biosciences, USA). Data were analyzed with FlowJo software (BD Biosciences, USA).

**Table 1:** Antibodies used for the characterization of immune cells and MSCs.

Target	Marker for	Clone	Company	Catalog number	Species of origin	Dilution
CD105	MSCs	REA794	Miltenyi Biotec	130‐112‐168	REAfinity^TM^	1:20
CD14	Monocytes	REA599	Miltenyi Biotec	130‐128‐771	REAfinity^TM^	1:100
CD15	Neutrophils	VIMC6	Miltenyi Biotec	130‐113‐489	mouse	1:100
CD19	B Cells	REA675	Miltenyi Biotec	130‐113‐646	REAfinity^TM^	1:100
CD34	Hematopoietic stem cells	REA1164	Miltenyi Biotec	130‐120‐515	REAfinity^TM^	1:100
CD45	Pan T cells	REA747	Miltenyi Biotec	130‐110‐632	REAfinity^TM^	1:100
CD73	MSCs	REA804	Miltenyi Biotec	130‐111‐909	REAfinity^TM^	1:20
CD90	MSCs	REA897	Miltenyi Biotec	130‐114‐859	REAfinity^TM^	1:20
HLA‐DR	MHC II complex	REA805	Miltenyi Biotec	130‐111‐789	REAfinity^TM^	1:100

### Gene Expression Analysis

The in vitro FH models were processed as shown in 4.11. After co‐cultivation experiments, the in vitro FH models were separated from the bone models and dissociated accordingly. Afterward, the cells were centrifuged for 3 min at 4 °C and 300 x *g* and resuspended in RLT buffer (Qiagen, Hilden, Germany) with 1% 2‐Mercaptoethanol (Serva Electrophoresis, Heidelberg, Germany). Total RNA was extracted using the RNeasy Mini Kit (Qiagen, Hilden, Germany) according to the manufacturer's instructions, and RNA concentration was determined using the Nanodrop ND‐1000 (Peqlab, VWR International, USA). RNA was stored at −80 °C until further processing.

cDNA was synthesized by reverse transcription using TaqMan Reverse Transcription Reagents (Applied Biosystems, Carlsbad, USA). qPCR was performed using the DyNAmo Flash SYBR Green qPCR Kit (Thermo Fisher Scientific, USA) and the QuantStudio 7 (Thermo Fisher Scientific, USA). Initial denaturation was for 7 min at 98 °C, followed by 55 cycles of 5 s at 98 °C, 7 s at 56 °C, and 9 s at 72 °C. The melting curve was analyzed by stepwise increasing the temperature from 50 °C to 98 °C every 30 s. All primers were designed with Primer3, verified with NCBI Primer‐BLAST, purchased from TIB Molbiol (Berlin, Germany), and verified by sequencing beforehand (**Table** **2**).

**Table 2:** Primer sequences.

Gene	Gene name	forward primer	reverse primer
*CXCR4*	C‐X‐C chemokine receptor type 4	GCATGACGGACAAGTACAGGCT	AAAGTACCAGTTTGCCACGGC
*EF1A*	Elongation factor 1‐alpha	GTTGATATGGTTCCTGGCAAGC	TTGCCAGCTCCAGCAGCCT
*IL13*	Interleukin 13	AGACCAGACTCCCCTGTGCA	TGGGTCCTGTAGATGGCATTG
*IL4*	Interleukin 4	CGGCAACTTTGTCCACGGA	TCTGTTACGGTCAACTCGGTG
*IL6*	Interleukin 6	TACCCCCAGGAGAAGATTCC	TTTTCTGCCAGTGCCTCTTT
*IL8*	Interleukin 8	GGACCCCAAGGAAAACTGG	CAACCCTACAACAGACCCACAC
*LDHA*	Lactate dehydrogenase A	ACCCAGTTTCCACCATGATT	CCCAAAATGCAAGGAACACT
*MMP2*	Matrix metalloproteinase‐2	GATACCCCTTTGACGGTAAGGA	CCTTCTCCCAAGGTCCATAGC
*MMP9*	Matrix metalloproteinase‐9	CCTGGAGACCTGAGAACCAATC	CCACCCGAGTGTAACCATAGC
*RUNX2*	Runt‐related transcription factor 2	TTACTTACACCCCGCCAGTC	TATGGAGTGCTGCTGGTCTG
*VEGFA*	Vascular endothelial growth factor A	AGCCTTGCCTTGCTGCTCTA	GTGCTGGCCTTGGTGAGG

Eukaryotic translation elongation factor 1 alpha 1 (*EF1A*) was used as the housekeeping gene because it has been previously reported to be stable for the experiments performed here. The 2^−ΔCt^ method was employed to analyze the gene expression data.

### In Vitro Micro‐Computed Tomography

The bone models were scanned at a nominal resolution of 5 µm using a SkyScan 1172 high‐resolution microCT (Bruker, Kontich, Belgium). The X‐ray tube voltage was set to 80 kV, and a 0.5 mm aluminum filter was employed to reduce beam hardening effects. The scan orbit covered 360° with a rotation step of 0.1 degrees. After scanning, reconstruction was performed using Bruker's GPU‐accelerated NRecon software. The total volume of interest (VOI) of the bone models was analyzed using CTAn software (Bruker, Kontich, Belgium). The threshold for bony tissue was set globally using the Otsu method and kept constant for all models.

### Immunofluorescence Staining

Immunofluorescence staining was performed in a dark, humid chamber at RT. First, slices were air‐dried and then rehydrated with PBS for 10 min. Unspecific binding sites were blocked with PBS containing 5% FCS for 30 min. The primary antibody was then diluted in PBS with 5% FCS and 0.1% Tween 20 (Qbiogene Inc., Carlsbad, USA) and incubated according to the manufacturer's instructions, followed by three washes with PBS containing 0.1% Tween 20. Next, the secondary antibody, diluted in PBS with 5% FCS and 0.1% Tween 20, was applied for 2 h. This was followed by three washes with PBS containing 0.1% Tween 20. Nuclei staining was performed using 4′,6‐diamidino‐2‐phenylindole (DAPI; 1 µg mL^−1^ in PBS with 5% FCS and 0.1% Tween 20) for 15 min. After ensuring the samples were bubble‐free, they were covered with FluoroMount (Sigma‐Aldrich, Munich, Germany) and imaged using the laser scanning fluorescence microscope LSM 710 (Carl Zeiss, Jena, Germany) using lasers of specific wavelengths. The primary and secondary antibodies used for immunofluorescence staining are listed in **Table** **3**.

**Table 3:** Primary and secondary antibodies used for immunofluorescence staining.

Target	Host	Clonality	Dye	Company	Catalog number	Dilution
ALKALINE PHOSPHATASE	mouse	monoclonal	–	Abcam	ab126820	1:200
COLLAGEN I	recombinant	monoclonal	–	Abcam	ab138492	1:200
COLLAGEN IV	recombinant	monoclonal	Alexa 647	Abcam	ab200430	1:200
OSTEOCALCIN	recombinant	monoclonal	Alexa594	Abcam	ab152232	1:200
OSTEOPONTIN	recombinant	monoclonal	Alexa488	Abcam	ab281819	1:200
C‐FOS	recombinant	monoclonal	–	Abcam	ab302667	1:200
ANTI‐MOUSE	goat	polyclonal	Alexa594	Thermo Fisher	A‐31573	1:500
ANTI‐GOAT	rabbit	polyclonal	Alexa488	Abcam	ab150077	1:500

### Statistical Analysis

Statistical analysis was performed using the GraphPad Prism V.8.4.1 software (GraphPad Software, San Diego, USA). Data are shown as box plots or bar charts. Statistical analyses were performed according to the data and are depicted for each figure in the respective legend. P‐values are indicated in the graphs with ^#^ *p* > 0.1, *p < 0.05, **p < 0.01, ***p < 0.001, ****p < 0.0001.

## Conflict of Interest

The authors declare that they have no conflict of interest.

## Author Contributions

M.P., T.G., A.D., and F.B. study design. M.P., A.D., J.P., M.T., and A.A. performed data collection and analysis. M.P., A.D., T.G., and F.B. drafted the manuscript. M.P., A.D., T.G., and F.B. performed data discussion and interpretation. M.P., A.D., F.B., P.H., T.G., and J.S. revised the manuscript. M.P., A.D., T.G., and F.B. contributed equally to this work.

## Supporting information



Supporting Information

Supporting Information

Supporting Information

## Data Availability

The data that support the findings of this study are available on request from the corresponding author. The data are not publicly available due to privacy or ethical restrictions.
