# Supplementary material for: A Modular Perfusion Bioreactor Platform for Simulating Bone Regeneration and Fracture Healing: Integrating Mechanical Loading and Dual Perfusion for Advanced In Vitro Models
Source: Adv Healthc Mater. 2025 Aug 15;14(32):e02492. doi: 10.1002/adhm.202502492 (PMC12716203; doi:10.1002/adhm.202502492)
Supplement: Supplementary file 1 — Supporting Information [file ADHM-14-0-s002.docx]

# Supplement

#
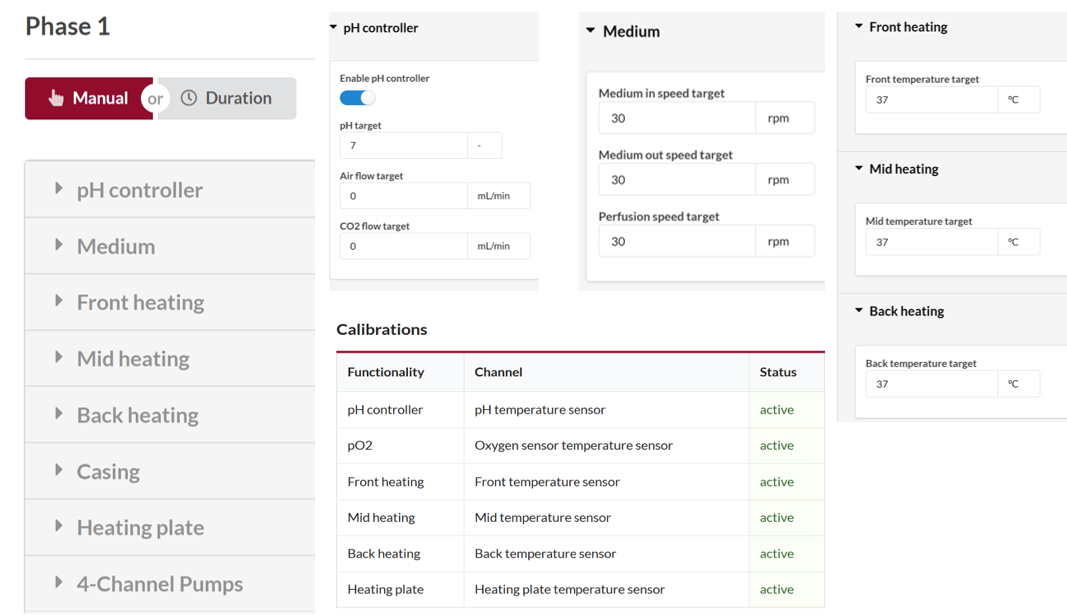


**Supplementary Figure 1: User interface for manual configuration of the modular bioreactor platform:** Screenshots of the control software during manual operation, illustrating the adjustable parameters for environmental and mechanical conditioning of the culture system. Parameters include pH control (target value and gas flow rates), medium perfusion (in/out flow and loop speed), and temperature control for three separate heating zones (front, mid, and back), each set to 37 °C. The calibration table indicates active status of all integrated temperature and oxygen sensors across corresponding modules, ensuring accurate feedback-based regulation.
