# Supplementary figures and images for: A Modular Perfusion Bioreactor Platform for Simulating Bone Regeneration and Fracture Healing: Integrating Mechanical Loading and Dual Perfusion for Advanced In Vitro Models
Source: Adv Healthc Mater. 2025 Aug 15;14(32):e02492. doi: 10.1002/adhm.202502492 (PMC12716203; doi:10.1002/adhm.202502492)

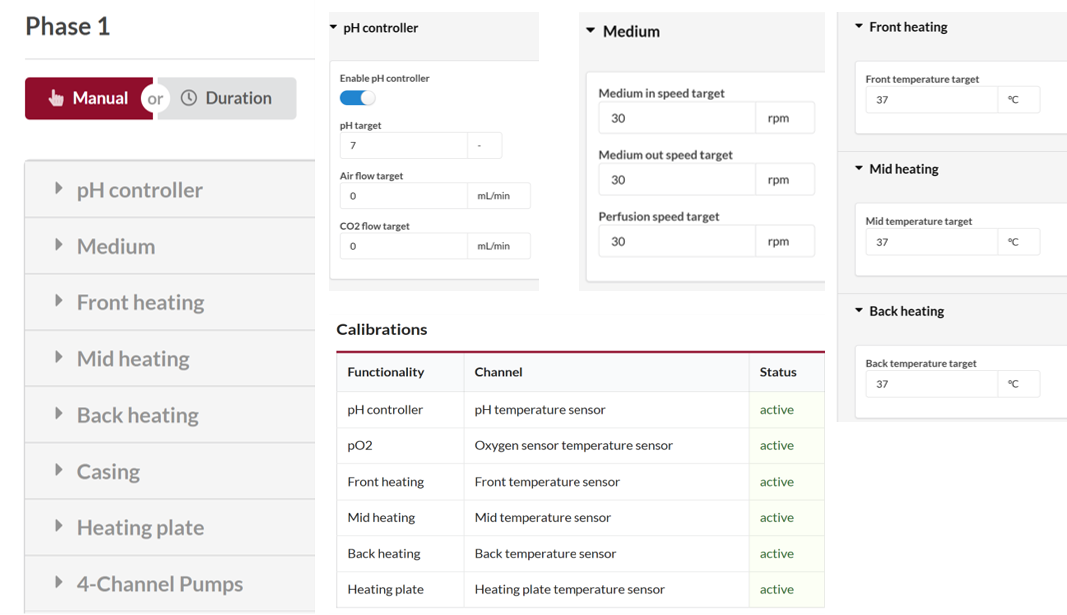

Supplement: Supplementary file 2 — Supporting Information [file ADHM-14-0-s001.png]
